# Environmental maternal exposures and the risk of premature birth and intrauterine growth restriction: The Generation Gemelli study protocol of newborn exposome

**DOI:** 10.1371/journal.pone.0317458

**Published:** 2025-01-16

**Authors:** Leonardo Villani, Angelo Maria Pezzullo, Roberta Pastorino, Alessandra Maio, Francesca Stollagli, Chiara Tirone, Marta Barba, Angela Maria Cozzolino, Denise Pires Marafon, Martina Porcelli, Annamaria Sbordone, Maria Letizia Patti, Anthea Bottoni, Angela Paladini, Simona Fattore, Domenico Marco Romeo, Ornella Parolini, Wanda Lattanzi, Guido Rindi, Luca Tamagnone, Marco Marazza, Maurizio Genuardi, Elisabetta Tabolacci, Eugenio Maria Mercuri, Antonio Chiaretti, Tina Pasciuto, Maurizio Sanguinetti, Vincenzo Valentini, Giovanni Scambia, Walter Ricciardi, Giovanni Vento, Antonio Lanzone, Stefania Boccia

**Affiliations:** 1 University Department of Life Sciences and Public Health, Università Cattolica del Sacro Cuore, Rome, Italy; 2 Department of Woman and Child Health and Public Health, Fondazione Policlinico Universitario A. Gemelli, IRCCS, Rome, Italy; 3 Unità Operativa Complessa di Neonatologia, Fondazione Policlinico Universitario A. Gemelli, IRCCS, Rome, Italy; 4 Biobanca di Ricerca per la Medicina Personalizzata, Fondazione Policlinico Universitario A. Gemelli, IRCCS, Rome, Italy; 5 Pediatric Neurology Unit, Fondazione Policlinico Universitario A. Gemelli, IRCCS, Rome, Italy; 6 Fondazione Policlinico Universitario A. Gemelli, IRCCS, Rome, Italy; 7 Pediatric Emergency Department, Fondazione Policlinico Universitario A. Gemelli, IRCCS, Rome, Italy; 8 Research Core Facility Data Collection G-STeP, Fondazione Policlinico Universitario A. Gemelli, IRCCS, Rome, Italy; 9 Department of Laboratory Sciences and Infectious Diseases, Fondazione Policlinico Universitario A. Gemelli, IRCCS, Roma, Italy; 10 Department of Diagnostic Imaging, Oncologic Radiotherapy and Hematology, Fondazione Policlinico Universitario A. Gemelli, IRCCS, Roma, Italy; University of Life Sciences in Lublin, POLAND

## Abstract

**Background:**

The study of women exposures and child outcomes occurring in the first 1,000 days of life since conception enhances understanding of the relationships between environmental factors, epigenetic changes, and disease development, extending beyond childhood and spanning the entire lifespan. Generation Gemelli is a recently launched case-control study that enrolls mother-newborns pairs in one of the largest university hospitals in Italy, in order to examine the association between maternal environmental exposures and intrauterine growth restriction (IUGR) and the risk of premature birth. The study will also evaluate the association of maternal exposures and the health and growth of infants and children up to 24 months of age.

**Methods:**

The study entails the set-up of a case-control study within a birth cohort. With approximately 4,000 annual deliveries, we aim to enroll 140 cases (newborns with IUGR and premature birth) and 280 controls per year, from September 2022. A comprehensive questionnaire will be used to gather information about various types of maternal environmental exposures before and during pregnancy. We will collect biological samples from both mothers and newborns (including vaginal swab, placenta sample, blood, saliva, meconium, and bronchoalveolar lavage fluid) at birth and within the early hours of the newborn’s life. We will perform laboratory examinations including dosage of heavy metals and essential elements, investigation of placental distress and fetal brain damage of biomarkers, analysis of microbiota and of DNA methylation profile. We will conduct clinical follow-up assessments in both cases and controls at months 12 and 24 and we will collect anthropometric data, feeding types with particular reference to breastfeeding and its duration, pediatric emergency room visits, hospitalizations, medication usage, known allergies, and neuropsychological development.

**Discussion:**

The Generation Gemelli case-control study holds the promise of significantly enhancing our comprehension of how maternal environmental exposures relate to the health of children and the broader population. The study of the exposome will provide insights into the relationships between environmental exposures, epigenetic changes and health outcomes during the first 1000 days of life and onward.

## Introduction

The health status, growth, and development of children are strongly influenced by the exposures to which they are affected from the periconceptional period through early life [1, 2]. In particular, environmental factors have an impact not only on childhood, but throughout the entire life course, with accumulating evidence of relationships between exposures in early life and the development of chronic diseases [2, 3], as well as neurological disorders, growth alterations and constitutional delay [4–6]. Special attention has recently been paid to the first 1000 days of life (the time spanning roughly between conception and the child’s second birthday), a critical period that entails rapid growth and development of organs and systems, primarily the brain, occurs [7, 8]. During this period, organs are particularly vulnerable and affected by exposure to a wide range of factors, which can be both positive and negative, and in many cases can be controlled and modified. During pregnancy, the mother and the fetus are intricately intertwined and interdependent, representing an inseparable constraint, where maternal exposures are reflected on the fetus, impacting its health, growth and development [2, 9–11]. Similarly, parents’ environmental exposures before conception and pregnancy may influence the health status of future children [12, 13].

The study of the child’s exposome, therefore, represents an important opportunity to understand the mechanisms of development and pathologies related to environmental factors, as childhood exposome includes exposures experienced from in utero until childhood, with short latency of associations between the exposure and the development of disease [14, 15]. The concept of exposome was developed in 2005 and encompasses all human environmental factors to which humans are exposed from conception onward [16]. Over time, this definition has been expanded [17–20] and has led to the identification of 3 overlapping domains that include all exposures: the external exposome that includes both exposures at individual (smoking, diet, physical activity, infections) and community level (air pollution and other climate factors, urban environment, social conditions), and the internal exposome, that includes biological factors such as hormone activities, inflammation, gut microflora, and oxidative stress, which could be identified by specific biomarkers and personal omics profiling (metabolomics, proteomics, transcriptomics and epigenomics).

Epidemiological studies provide an opportunity to study newborns’ exposome evaluating the association between maternal environmental exposures and neonatal and child outcomes during the first 1000 days of life [14], with relevant implications in public health [20]. These studies, indeed, allow to move from a “one-exposure-one-health-effect” to the measurement and identification of widespread exposure effect sizes on health outcomes, in a holistic and systemic approach [17–19].

The aim of our study is to set-up a case-control study within the birth cohort of newborns assisted at the Fondazione Policlinico Universitario A. Gemelli IRCCS (FPG), in Rome, Italy, in order to assess the association between maternal environmental exposures and intrauterine growth restriction (IUGR) and the risk of premature birth. The secondary objective is to evaluate the association between maternal exposures and fetal and neonatal growth and well-being, and the physical, cognitive, and behavioral growth and development of the children.

## Material and methods

### Study design, population and inclusion criteria

In this case-control study, we enroll mother-newborns pairs during pregnancy and at delivery and prospectively follow the children during the first 24 months of life. Biological samples from the mother and newborns are collected to assess internal exposome; in addition, a questionnaire investigating the habits and lifestyles of mothers before and during pregnancy is administered to assess the association between environmental exposures and the health status of fetus.

Inclusion criteria are (I) being resident for at least two years in the city of Rome, Italy; (II) being assisted and give birth at FPG and (III) meet the definition of case or control.

**Fig 1:**
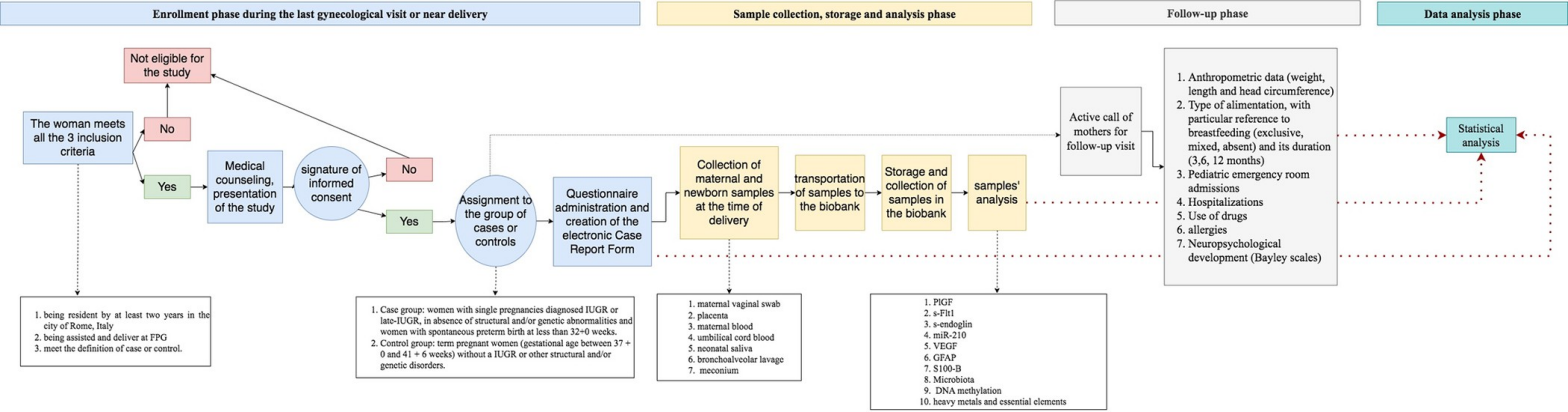
Flowchart of the process, from patient enrollment to data analysis.

Fig 1 shows the different phases of the study, from mothers’ enrollment to data analysis.

### Case and control definition

Cases are defined as (I) women with single pregnancies diagnosed with defined late intrauterine growth restriction (≥32 weeks of gestational age), according to the definition of Gordijn et al [21], in the absence of structural and/or absence of evident organs malformations, and (II) women with spontaneous preterm birth at less than 32+0 weeks of gestational age, with the inclusion of preterm births up to this threshold aligning with the late preterm birth classification recommended by the American College of Obstetricians and Gynecologists (ACOG) and the Society for Maternal-Fetal Medicine (SMFM) [22]. The control group is represented by full term pregnant women (gestational age between 37 + 0 and 41 + 6 weeks) without a IUGR or other structural and/or genetic disorders. Patients with preeclampsia, but without signs of IUGR, will also be included in the control group. The method of delivery will not be considered a factor in the inclusion criteria for either the case or control groups.

### Recruitment

**Fig 2:**
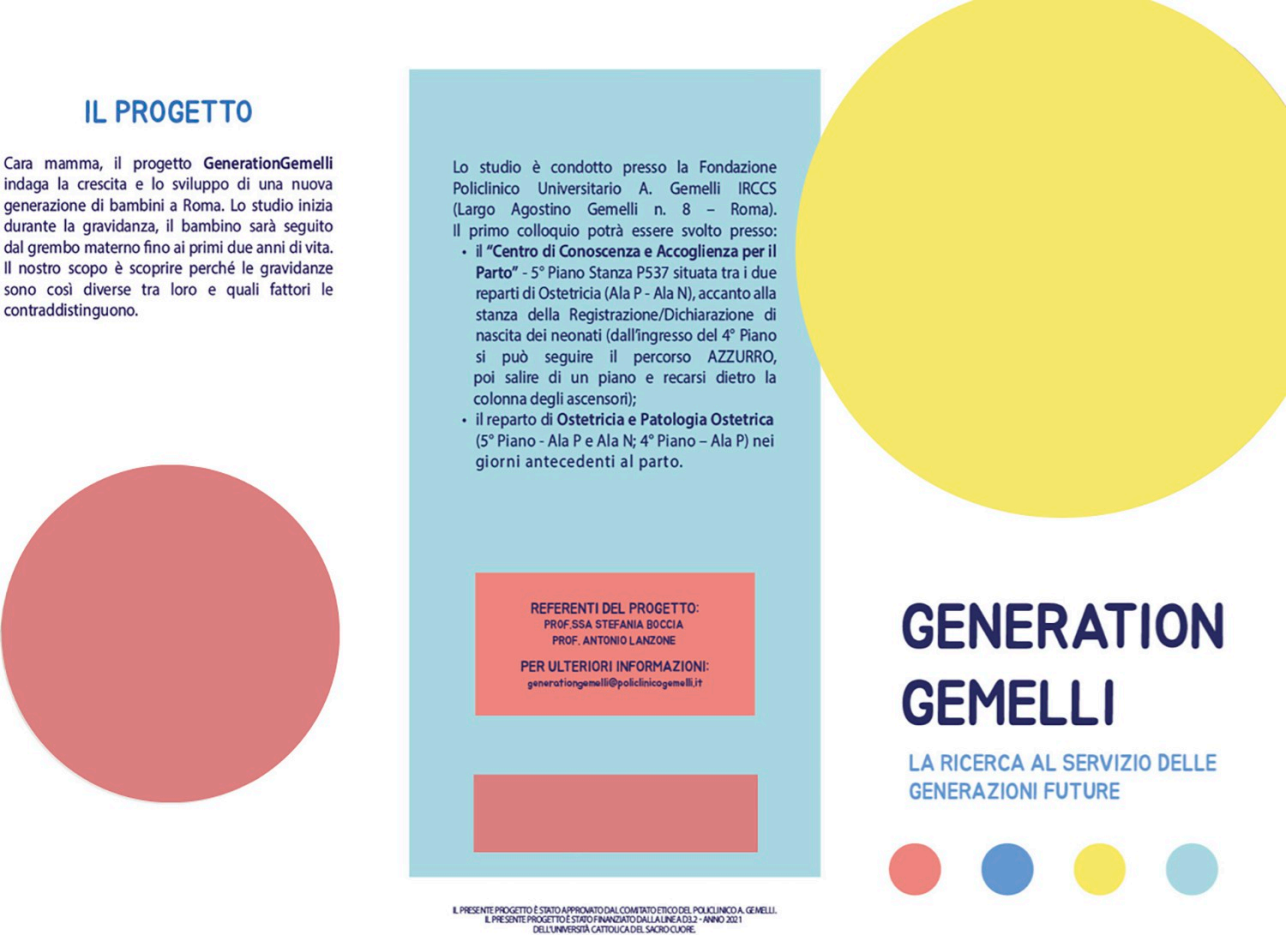
Information materials distributed to women before and after enrollment.

After approval by the Ethics Committee of Policlinico Universitario A. Gemelli—IRCCS (protocol number ID4975) on 19^th^ July 2022, recruitment started on 15^th^ September 2022 and is planned to continue until 2024. All pregnant women and their partners are invited to participate in the study during the last gynecological routine visit or at admittance to the hospital for delivery. During the visit, the study is explained by medical staff, who verify women’s eligibility and obtain informed consent signatures. Personnel from the Obstetrics Unit, specifically trained for the project, distribute informational materials (Fig 2) and supervise the completion of the questionnaire, addressing any doubts or questions from participants while ensuring that the responses remain uninfluenced. Each woman is given a unique identification number, which will then be identical to that for the biological samples collected for the newborn, in order to allow the tracking of the mother-newborn pair. Moreover, this procedure ensures the pseudo-anonymization of data, confidentiality and personal data protection.

### Follow-up

Mothers are informed of follow-up visits during the enrollment phase. They will then be contacted by telephone (via text message, phone call, or email if no response is received) at months 12 and 24 in order to perform an assessment of the child’s health status. Specifically, the follow-up visit involves a clinical examination with anthropometric data collection (weight, length and head circumference with associated Z scores) with specific attention to the type of alimentation and breastfeeding and the development of allergies. Moreover, pediatric emergency room admissions and hospitalizations and use of medications and drugs will be investigated. Finally, a neuropsychological development evaluation will be performed with the Bayley scales [23]. However, if the visit cannot be conducted at the 12th and 24th months, the baby will unfortunately be lost to follow-up, and a change of residence could increase the likelihood of this.

### Questionnaire

The Pregnancy Exposures Questionnaire (see S1 Questionnaire for the full document) is administered by physicians after the women had read the information sheet and given the informed consent. The questionnaire, administered in the Italian language, has been adapted to our context on the basis of the Intergrowth-21^st^ project and the Japan Environment and Children Study [24, 25]. The questionnaire is divided into 8 sections (Table 1), including general and specific information about exposures in pregnancy, for a total of 76 questions, with a time to compile of approximately 10–15 minutes. For each question there are several response options according to a frequency scale (e.g., never, rarely, often, always, or every day, 3/4 times a week, once a week, once a month, less than once a month).

**Table 1:** The eight sections of the Pregnancy Exposures Questionnaire.

Section	Topic
Section 1	Demographic and pregnancy information
Section 2	Lifestyles before and during pregnancy
Section 3	Characteristics of the house
Section 4	Environmental characteristics of the workplace
Section 5	Environmental exposures in daily life and domestic house
Section 6	Types of food consumed and possibility of access to food
Section 7	Vaccination intentions and attitudes
Section 8	Type of job and occupation

### Collection and storage of biologic samples

Biological samples from the mother and newborn are collected at the time of delivery and during the first hours of life (saliva, meconium and bronchoalveolar lavage fluid–BALF). Subsequently, they are processed and cryopreserved at the Biobank for Research in Personalized Medicine of the FPG.

Specifically, maternal samples are collected at the time of delivery and include a vaginal swab (immediately transported to biobank and stored at -80°C), a placenta sample (immediately transported to biobank and stored at -80°C), and a blood sample that will be centrifuged to obtain the plasma fraction (dispensed in 3 aliquots of 1 ml), which will be collected and stored at -80°C for subsequent analysis. The remaining samples are processed for the isolation of peripheral blood mononuclear cells (PBMCs) (dispensed in 3 aliquots, approximately 2–3 million cells per vial), which will be stored at -196°C.

The sample of the newborn collected during delivery includes umbilical blood that is centrifuged to obtain the plasma fraction (dispensed in 3 aliquots of 1 ml), which will be collected and stored at -80°C. The residual sample of blood will be processed for the isolation of PBMCs (dispensed in 3 aliquots, approximately 2–3 million cells per vial), which will also be stored at -196°C. In addition, a sample of the first meconium in 30 ml stool tubes, a sample of saliva obtained in the first 24 hours of life in 2 ml cryovials, and a BALF sample collected only from intubated and ventilated newborns at birth in 30 ml tubes are collected and immediately transported to the biobank for storage at -80°C.

For both maternal and newborn blood samples, if the required aliquots are not reached, blood is dedicated only for isolation of PBMCs, while plasma is not collected. If a sample is deemed unsuitable (e.g., due to blood clots or improper transport temperature), it will not be processed. In cases where it is feasible (e.g., maternal blood collection), a second sample will be obtained.

### Laboratory analysis

The following investigations are carried out on the collected samples or subsets of samples: dosage of heavy metals and essential elements in the blood of the mother and the newborn; analysis of biomarkers of placental distress in maternal blood such as angiogenic placental growth factor (PlGF), soluble FMS-like tyrosine kinase-1 (s-Flt1), s-endoglin, miR-210, vascular endothelial growth factor (VEGF); investigation of fetal brain damage biomarkers, both in maternal blood and newborn saliva, such as glial fibrillary acidic protein (GFAP), S100-B; analysis of microbiota and of DNA methylation profile. In both maternal (vaginal swabs, placenta) and neonatal (BALF, meconium) samples, bacterial DNA extraction will be performed for microbiota analysis in a strictly controlled level-2 biological safety workplace using Danagene Microbiome DNA kits (Danagen-Bioted) according to manufacturers’ instructions. DNA will be fluorometrically quantified (Qubit dsDNA high-sensitivity assay, Thermo Fisher Scientific), and then subjected to the 16S rRNA V3-V4 region amplification. The resulting amplicons will be purified using Agencourt AMPure XP beads (Beckman Coulter) and indexed using the Nextera XT Index kit (Illumina).

The selection of biomarkers for analysis may be modified and updated based on new literature evidence and collaborations and networking with other projects and birth cohorts.

### Outcomes of interests, sample size and minimum detectable effect size

The measures of the primary outcome of interest (being case or control) for this study are the (exposure) odds ratios (ORs). Secondary outcomes of interest are related to the physical and neuropsychological developments of the cohorts of cases and controls. Out of approximately 4000 annual deliveries performed in the hospital, which serves women from all areas of Rome, 200 are estimated to be classifiable as cases. Controls will be enrolled in a 2:1 ratio with cases, randomly selected from the cohort of pregnant women afferent to the hospital and matched for calendar of birth and maternal age with an interval of 5 years. Assuming a 70% response rate, we expect to enroll 140 cases and 280 controls per year. A 20% missing data rate in biological sample collection due to lack of specific consent or logistical difficulties in retrieval at the time of delivery is expected. Table 2 reports the minimum detectable effect size of the primary outcome of interest expressed as OR and absolute difference of proportions according to the prevalence of exposure, with a power = 80% and type 1 error = 5%.

**Table 2:** Minimum detectable effect size expressed as odds ratio (OR) and absolute difference of proportions according to exposure prevalence (power = 80%; type 1 error: 5%).

Proportion of exposed in the control group (%) (n_cases_ = 280; n_controls_ = 560)	Minimum OR by risk factor (minimum difference between the proportions of exposures in the two groups, %)	Minimum OR by protective factor (minimum difference between the proportions of those exposed in the two groups, %)
50	1.27 (10)	0.79 (10)
25	1.30 (9)	0.75 (8)
10	1.44 (7)	0.65 (5)
5	1.62 (6)	0.53 (3)
3	1.81 (5)	0.42 (3)
1	2.49 (3)	0.14 (NC)

### Statistical analysis: Descriptive analysis and multivariable analysis

All data are monitored through analysis of ranges, distributions, means, standard deviations and outliers. Outliers and missing data will be compared with the original measurements. Characteristics of study participants will be presented in tables with mean and standard deviation (normally distributed continuous variables), median and interquartile range (non-normally distributed continuous variables), absolute frequency and percentage (categorical variables). We will perform inferential analysis to compare means (Welch t or Mann-Whitney U test, as appropriate) and frequencies among the case and control groups (chi-square, Fischer’s exact, Kruskal-Wallis, ANOVA, as appropriate). Univariable and multivariable regression models will be performed to quantify the effect, in the form of odds ratio/hazard ratio, of the independent variables on the primary and secondary outcomes of interest.

### Data management

Data processing takes place according to European legislation on General Data Protection Regulation (GDPR—2016/679). Pseudo-anonymized data are collected and managed using REDCap electronic data capture tools hosted at Fondazione Policlinico Universitario A. Gemelli, IRCCS, a secure web-based application designed to support data capture for research studies [26]. A dedicated electronic Case Report Form (eCRF) has been designed according to Good Clinical Practice requirements, and its development included annotated CRF design according to protocol, database setup and validation, and edit checks programming. All data from women enrollment to the end of the follow-up are collected. Data from the questionnaire are integrated with data from the medical records obtained during gynecological examinations, hematochemical analysis and diagnostic examination (such as ultrasound) conducted during pregnancy, and biological samples collected at the time of delivery. Such a tool, therefore, allows for the integration of all data related to the woman and the newborn. Data are entered into the eCRF in a truthful, accurate and timely manner. The investigator is responsible to ensure that the eCRF is properly and completely filled in. All data are ALCOA+ criteria compliant [27] and are maintained in confidence and protected. Only personnel officially registered as study investigators or data managers, who have received specific training, could access with a multifactorial authentication the web platform and manage data.

### Ethics approval and consent to participate

The study was approved on 19^th^ July 2022 by the Ethics Committee of Policlinico Universitario A. Gemelli—IRCCS, protocol number ID4975. Participation in the study is voluntary and non-compensated, and all participants must sign and provide informed consent prior to enrollment. Signing the informed consent also includes authorization for anonymous publication in scientific journals. Participants may withdraw from the study at any time.

## Discussion

The objectives of this case-control study are to assess the association between maternal environmental exposures and the risk of intrauterine growth restriction and premature birth and between the mother’s environmental exposures and the neonatal-child growth and well-being during the first 1000 days of life.

The study of the newborn’s exposome, starting from the uterine period until the first two years of life, allow to identify some possible associations underlying many chronic diseases that appear during childhood and onward [14], improving the understanding of the effect of exposures on health outcomes of populations. While the impact of many early environmental exposures on health has been already studied [28–30], more evidence is needed on the cumulative effect of multiple exposures and their impact, relationships and causal mechanisms with epigenetic changes, including DNA methylation, post-translational histone modifications and non-coding RNA expression [31].

Indeed, the possibility of studying these phenomena with a mixed approach, thus relying on both the collection of anamnestic data through questionnaires and medical history and the collection of biological samples from the mother and the newborn, in addition to clinical follow-up at months 12 and 24, represents a great opportunity to study the association between environmental exposures and human health in a systemic and holistic way [32, 33].

In this context, collaboration with other European and Italian birth cohorts is foreseen. In fact, there are currently numerous examples of prospective birth cohort studies [34], even in Italy [35–40], and close collaboration with these projects is planned to share data, information, and research methodologies. For this reason, the Generation Gemelli cohort has been included within a national project that involves the creation of a network of nine birth cohorts that will be active in four main domains: data harmonization (to make each cohort’s results comparable), georeferencing (to obtain accurate information on maternal exposures), shared protocols for laboratory analysis, and information and educational activities, both of health professionals and citizens.

We expect the study to encounter some limitations that will need to be recognized and considered in the interpretation of the results. One of the limitations will be the possible resistance of some women to be recruited, which could introduce a potential selection bias, as women who refuse to participate may differ in characteristics or experience from those who accept. Another concern is related to the loss of follow-up. However, efforts have been made to minimize these difficulties, creating a strong trust-based relationship between physicians and women. Moreover, all the strategies to encourage follow-up retention (email, phone calls, text messages) will be adopted. Finally, this study involves several professionals with different backgrounds, thus requiring a multidisciplinary approach. In this context, another potential limitation could be the operational challenge of establishing a cohesive workflow based on the coordination and alignment of efforts. However, regular in-person meetings (weekly and then monthly) have ensured the identification of a valuable working group that discusses operational problems and solutions, while the creation of a standardized operational flow ensures efficiency appropriateness and quality.

## Conclusions

The Generation Gemelli study will contribute to the understanding of early-life risk factors for newborn-child and adult health, from in utero life to the first two years of life. Using a prospective approach, we will be able to assess the children’s exposome including both external and internal environmental exposures and effects. Understanding the effects of environmental exposures on human health has a profound impact in public health, informing policymakers to adopt possible targeted interventions on selected populations to improve quality of life and reduce risk associated with harmful exposures.

## Supporting information

S1 Questionnaire Pregnancy exposures questionnaire. (DOCX)
