# Supplementary material for: Environmental maternal exposures and the risk of premature birth and intrauterine growth restriction: The Generation Gemelli study protocol of newborn exposome
Source: PLoS One. 2025 Jan 16;20(1):e0317458. doi: 10.1371/journal.pone.0317458 (PMC11737710; doi:10.1371/journal.pone.0317458)
Supplement: S1 Questionnaire — (DOCX) [file pone.0317458.s001.docx]

**Pregnancy Exposures Questionnaire**

[**SECTION 1. Demographic and pregnancy information** 2](#_Toc141983967)

[**SECTION 3. Housing characteristics** 8](#_Toc141983968)

[**SECTION 4. Environmental characteristics of the workplace** 11](#_Toc141983969)

[**SECTION 5. Environmental exposures in daily life and housing** 13](#_Toc141983970)

[**SECTION 6. Types of food consumed and accessibility of food** 15](#_Toc141983971)

[**SECTION 7: Vaccination intentions and habits** 19](#_Toc141983972)

[**SECTION 8: Type of job and occupation** 21](#_Toc141983973)

**SECTION 1. Demographic and pregnancy information**

1. Age: __
2. Citizenship: _____
3. Marital Status: ______
4. Educational level:

| **Educational level** | **No** | **Ongoing** | **Completed** |
| --- | --- | --- | --- |
| - Elementary School Graduation |  |  |  |
| - Middle School Graduation |  |  |  |
| - High School Graduation |  |  |  |
| - Bachelor Degree |  |  |  |
| - First Level Master Degree |  |  |  |
| - Master degree |  |  |  |
| - Second Level Master Degree |  |  |  |
| - Ph.D or Residency |  |  |  |

1. What is your current job: ____
2. Have you kept your job during your pregnancy?

- Yes
- No

1. Number of pregnancies prior to this pregnancy: _____
2. Number of children prior to this pregnancy: ____
3. Age of children: ____
4. Have you had any complications in previous pregnancies?

- Yes (please specify)
- No

1. Presence of child-dependent illnesses?

- Yes (please specify)
- No

1. At what week did you find out you were pregnant?: _____
2. Height (cm): ____
3. Weight before pregnancy: _____
4. Current weight: _____
5. How did you distribute maternity leave?

- Months before childbirth: ____
- Months after childbirth: ____
- I didn't use maternity leave **SECTION 2.** **Lifestyles before and during pregnancy**

***- Cigarette smoke***

1. Exposure to second-hand smoke at home:

- Very frequently
- Frequently
- Almost never
- Never

1. Exposure to second-hand smoke at job:

- Very frequently
- Frequently
- Almost never
- Never

1. Cigarette smoke before and during pregnancy:

| **CIGARETTE SMOKE** | **Cigarettes** | | **Heated tobacco cigarettes (Iqos, Glo etc.)** | | **Electronic cigarettes** | |
| --- | --- | --- | --- | --- | --- | --- |
| **BEFORE PREGNANCY** | **Yes** | **No** | **Yes** | **No** | **Yes** | **No** |
| Were you smoking before pregnancy? |  |  |  |  |  |  |
| How many cigarettes/daily did you smoke before pregnancy? |  |  |  |  |  |  |
| How many years had you been smoking before pregnancy? |  |  |  |  |  |  |
| **DURING PREGNANCY** | **Yes** | **No** | **Yes** | **No** | **Yes** | **No** |
| Are you smoking during pregnancy? |  |  |  |  |  |  |
| Did you quit smoking? |  |  |  |  |  |  |
| - when you learned about the pregnancy |  |  |  |  |  |  |
| - within 3 months of the start of pregnancy* |  |  |  |  |  |  |
| - after 3 months of the start of pregnancy* |  |  |  |  |  |  |
| Are you continuing to smoke during pregnancy? |  |  |  |  |  |  |
| - occasionally (not every day) |  |  |  |  |  |  |
| - every day, but less than before |  |  |  |  |  |  |
| - as before |  |  |  |  |  |  |
| - more than before |  |  |  |  |  |  |

* or specify pregnancy's month in which you quitted smoking

***- Alcohol***

1. Alcohol before and after pregnancy:

| **ALCOHOL CONSUMPTION** | **Wine** | | **Beer** | | **Hard liquor** | |
| --- | --- | --- | --- | --- | --- | --- |
| **BEFORE PREGNANCY** | **Yes** | **No** | **Yes** | **No** | **Yes** | **No** |
| Were you drinking alcohol before pregnancy? |  |  |  |  |  |  |
| How many glasses/week did you drink before pregnancy? |  |  |  |  |  |  |
| How many years had you been drinking before pregnancy? |  |  |  |  |  |  |
| **DURING PREGNANCY** | **Yes** | **No** | **Yes** | **No** | **Yes** | **No** |
| Are you drinking during pregnancy? |  |  |  |  |  |  |
| Did you stop drinking? |  |  |  |  |  |  |
| - when you learned about the pregnancy |  |  |  |  |  |  |
| - within 3 months of the start of pregnancy* |  |  |  |  |  |  |
| - after 3 months of the start of pregnancy* |  |  |  |  |  |  |
| Are you continuing to smoke during pregnancy? |  |  |  |  |  |  |
| - occasionally (not every day) |  |  |  |  |  |  |
| - every day, but less than before |  |  |  |  |  |  |
| - as before |  |  |  |  |  |  |
| - more than before |  |  |  |  |  |  |

* or specify month of pregnancy in which you stopped drinking

- ***Physical activity***

1. Physical activity before pregnancy:

| **PHYSICAL ACTIVITY BEFORE PREGNANCY** | | | | |
| --- | --- | --- | --- | --- |
| **Did you use to do these physical activities?:** | **Never** | **1 time a week** | **2-3 times a week** | **Every day** |
| - Walking outdoors (at least 20') |  |  |  |  |
| - Cycling |  |  |  |  |
| - Gardening |  |  |  |  |
| - Going to the gym |  |  |  |  |
| - Engaging in competitive physical activity |  |  |  |  |
| - Household activities (washing, sweeping, ironing, etc.) |  |  |  |  |

1. Physical activity during first pregnancy's trimester:

| **PHYSICAL ACTIVITY DURING FIRST PREGNANCY'S TRIMESTER** | | | | |
| --- | --- | --- | --- | --- |
| **Did you use to do these physical activities?:** | **Never** | **1 time a week** | **2-3 times a week** | **Every day** |
| - Walking outdoors (at least 20') |  |  |  |  |
| - Cycling |  |  |  |  |
| - Gardening |  |  |  |  |
| - Going to the gym |  |  |  |  |
| - Engaging in competitive physical activity |  |  |  |  |
| - Household activities (washing, sweeping, ironing, etc.) |  |  |  |  |

1. Physical activity during second pregnancy's trimester:

| **PHYSICAL ACTIVITY DURING SECOND PREGNANCY'S TRIMESTER** | | | | |
| --- | --- | --- | --- | --- |
| **Did you use to do these physical activities?:** | **Never** | **1 time a week** | **2-3 times a week** | **Every day** |
| - Walking outdoors (at least 20') |  |  |  |  |
| - Cycling |  |  |  |  |
| - Gardening |  |  |  |  |
| - Going to the gym |  |  |  |  |
| - Engaging in competitive physical activity |  |  |  |  |
| - Household activities (washing, sweeping, ironing, etc.) |  |  |  |  |

1. Physical activity during third pregnancy's trimester:

| **PHYSICAL ACTIVITY DURING THIRD PREGNANCY'S TRIMESTER** | | | | |
| --- | --- | --- | --- | --- |
| **Did you use to do these physical activities?:** | **Never** | **1 time a week** | **2-3 times a week** | **Every day** |
| - Walking outdoors (at least 20') |  |  |  |  |
| - Cycling |  |  |  |  |
| - Gardening |  |  |  |  |
| - Going to the gym |  |  |  |  |
| - Engaging in competitive physical activity |  |  |  |  |
| - Household activities (washing, sweeping, ironing, etc.) |  |  |  |  |

**SECTION 3. Housing characteristics**

1. Residence type:

- apartment in a condominium
- detached home

| **HOUSING** | **< 1 year** | **1-3 years** | **3-5 years** | **5-10 years** | **10-20 years** | **>20 years** | **Don’t know** |
| --- | --- | --- | --- | --- | --- | --- | --- |
| - How many years has it been since the construction of the dwelling? (age of dwelling) |  |  |  |  |  |  |  |
| - How many years has it been since the last dwelling renovation? |  |  |  |  |  |  |  |
| - Number of years lived in current dwelling |  |  |  |  |  |  |  |

1. Presence of mold on the house's walls:

- Yes (please specify the area of the house)
- No

1. Presence of domestic animals:

- Yes (please specify type of animal)
- No

1. Presence of cooling air conditioners:

- Yes
- No

1. Presence of heating systems:

- Yes
- No

1. Type of heating:

- Centralized
- Autonomous
- Mixed

1. Type of power supply of heating systems:

- Gas
- Electricity
- Heat pump
- Kerosene
- Pellets
- Coal
- Petroleum

1. Frequency of household cleaning (dusting):

- Every day
- 2-4 times a week
- 1 time a week
- 1-2 times a month
- A few times a year

1. Frequency of household cleaning (floor washing):

- Every day
- 2-4 times a week
- 1 time a week
- 1-2 times a month
- A few times a year

1. Frequency of mattress sanitation and sanitization procedures:

- 1 time a week
- 1-2 times a month
- 4 times a year
- 2 times a year
- 1 time a year

1. Frequency of pillow sanitation and sanitization procedures:

- 1 time a week
- 1-2 times a month
- 4 times a year
- 2 times a year
- 1 time a year

1. Use of mite-proof covers for bed sheets:

- Yes
- No

1. Dwelling is located near agricultural sites:

- Yes (within 500 meters)
- Yes (within 1500 meters)
- No

1. Dwelling is located near urban roads with at least two lanes in each direction (e.g., ring road, junctions) or major thoroughfares (e.g., highways):

- Yes (within 500 meters)
- Yes (within 1500 meters)
- No

1. Dwelling is located near industrial sites:

- Yes (within 500 meters)
- Yes (within 1500 meters)
- No

1. Air in the outdoor environment is difficult to breathe:

- Very often
- Often
- Sometimes
- Never

1. Air in the outdoor environment makes eyes burn:

- Very often
- Often
- Sometimes
- Never

1. Sources of loud noise are present near the dwelling:

- Very often
- Often
- Sometimes
- Never

1. Garbage is present on the road:

- Very often
- Often
- Sometimes
- Never

**SECTION 4. Environmental characteristics of the workplace**

**(Information about workplace’s characteristics, with related exposures)**

1. Do you have to lift weights > 20 kg?:

- Very often
- Often
- Almost never
- Never

1. Work environment is noisy:

- Yes
- No

1. Working frequency:

| **Working frequency:** | **>5 times a week** | **1-4 times a week** | **1-3 times a month** | **Never** |
| --- | --- | --- | --- | --- |
| - Between 10 p.m. and sunrise before pregnancy |  |  |  |  |
| - In a hot place that causes sweating before pregnancy |  |  |  |  |
| - Between 10 p.m. and sunrise during pregnancy |  |  |  |  |
| - In a hot place that causes sweating during pregnancy |  |  |  |  |
| - Frequency of using a tool or driving a vehicle with a strong vibration |  |  |  |  |

1. Frequency of manipulation/contact:

| **Frequency of manipulation/contact with:** | **Every day** | **1-6 times a week** | **1-3 times a month** | **Never** |
| --- | --- | --- | --- | --- |
| - anticancer drugs |  |  |  |  |
| - lead |  |  |  |  |
| - cadmium, arsenic, chromium |  |  |  |  |
| - mercury |  |  |  |  |
| - formaldehyde and/or formalin |  |  |  |  |
| - microorganisms |  |  |  |  |
| - anesthetic drugs |  |  |  |  |
| - photocopying machines/lasers |  |  |  |  |
| - radioactive substances, radiation |  |  |  |  |
| - disinfectants |  |  |  |  |
| - cosmetic products |  |  |  |  |
| - organic solvents and/or paints |  |  |  |  |
| - organic solvents (ethanol, benzene, acetone) |  |  |  |  |
| - kerosene, gasoline, petroleum |  |  |  |  |
| - insecticides, pesticides, herbicides |  |  |  |  |

**SECTION 5. Environmental exposures in daily life and housing**

1. Frequency of gasoline manipulation/contact (filling stations):

- Every day
- 4-6 times a week
- 2-3 times a week
- 1 time a week
- 1-3 times a month
- Never

1. Use of room perfumers:

- Yes
- No

1. Use of mothproofing for clothes in the closet:

- Yes
- No

1. Use of insecticides:

- Yes
- No

1. Frequency of using insecticides (mosquito repellents, fly repellents, etc.):

- Every day
- 1 time a week
- 1-3 times a month
- < 1 time a month

1. Frequency of personal deodorant use:

- Very often
- Often
- Rarely
- Never

1. Frequency of cosmetics use:

- Very often
- Often
- Rarely
- Never

1. Frequency of hair colorants and dyes use:

- Very often
- Often
- Rarely
- Never

**SECTION 6. Types of food consumed and accessibility of food**

1. Did you perform a specific diet before pregnancy?

- Yes (specify type, e.g., vegetarian, high-calorie, low-calorie, high-protein, low-protein)
- No

1. Did you perform a specific diet during pregnancy?

- Yes (specify type, e.g., vegetarian, high-calorie, low-calorie, high-protein, low-protein)
- No

1. Type of food consumed:

| **Eating:** | **< 1 time a week** | **1-2 times a week** | **3-4 times a week** | **5-6 times a week** | **Every day** |
| --- | --- | --- | --- | --- | --- |
| - at restaurant |  |  |  |  |  |
| - at cafeteria |  |  |  |  |  |
| - at fast-food |  |  |  |  |  |
| - prepackaged foods (e.g. lunch boxes) |  |  |  |  |  |
| - frozen foods |  |  |  |  |  |
| - foods stored in aluminum cans or tins |  |  |  |  |  |

1. Consumption of food BEFORE pregnancy:

| **Consumption of:** | **Never** | **1-4 portion/week** | **5-9 portion/week** | **10-13 portion/week** | **≥14 portion/week** |
| --- | --- | --- | --- | --- | --- |
| Red meats (hamburgers, patties, beef, veal, pork) |  |  |  |  |  |
| Fruits (one serving about 110g) |  |  |  |  |  |
| Vegetables both cooked and raw (one serving about 70-100 grams) |  |  |  |  |  |
| White meats (chicken, rabbit, turkey) | NO | | Yes, how often (monthly)? | | |
| Cold cuts and sausages (sausage, salami, ham, mortadella, bacon) | NO | | Yes, how often (monthly)? | | |
| Fish | NO | | Yes, how often (monthly)? | | |
| Fritters | NO | | Yes, how often (monthly)? | | |
| Legumes (one serving about 70 grams) | NO | | Yes, how often (monthly)? | | |
| Do you consume barbecued or grilled meat/fish? | NO | | Yes, how often (monthly)? | | |
| Do you normally add salt to the diet (in addition to what is on the plate)? | NO | | YES | | |
| Are you in the habit of eating outside of main meals? | NO | | Yes, with what frequency (excluding the top 3)? | | |

1. Consumption of food DURING pregnancy:

| **Consumption of:** | **Never** | **1-4 portion/week** | **5-9 portion/week** | **10-13 portion/week** | **≥14 portion/week** |
| --- | --- | --- | --- | --- | --- |
| Red meats (hamburgers, patties, beef, veal, pork) |  |  |  |  |  |
| Fruits (one serving about 110g) |  |  |  |  |  |
| Vegetables both cooked and raw (one serving about 70-100 grams) |  |  |  |  |  |
| White meats (chicken, rabbit, turkey) | NO | | Yes, how often (monthly)? | | |
| Cold cuts and sausages (sausage, salami, ham, mortadella, bacon) | NO | | Yes, how often (monthly)? | | |
| Fish | NO | | Yes, how often (monthly)? | | |
| Fritters | NO | | Yes, how often (monthly)? | | |
| Legumes (one serving about 70 grams) | NO | | Yes, how often (monthly)? | | |
| Do you consume barbecued or grilled meat/fish? | NO | | Yes, how often (monthly)? | | |
| Do you normally add salt to the diet (in addition to what is on the plate)? | NO | | YES | | |
| Are you in the habit of eating outside of main meals? | NO | | Yes, with what frequency (excluding the top 3)? | | |

1. In the last 12 months, have you found that the food purchased was not enough and there was no money to buy more?

- Never
- Sometimes
- Often
- Don't know

1. In the last 12 months have you been able to eat nutritionally balanced meals (including pasta, rice, meat, fish, fruits, vegetables)?

- Never
- Sometimes
- Often
- Don't know

1. In the last 12 months, has a family member reduced the size of a meal or skipped a meal because there was not enough money to buy food?

- Yes
- No
- Don’t know

1. How often did it happen?

- Almost every month
- A few months, but not every month
- Only 1 or 2 months
- Don't know

1. In the last 12 months, have you eaten less than what you think was fair due to lack of money?

- Yes
- No
- Don’t know

1. In the last 12 months, have you ever felt hungry but did not eat because there was not enough money for food?

- Yes
- No
- Don’t know

# **SECTION 7: Vaccination intentions and habits**

1. Vaccination:

| **Are you intent on vaccinating your child for vaccinations** | **not at all agree** | **in partial disagreement** | **neither agree nor disagree** | **partially agree** | **totally agree** |
| --- | --- | --- | --- | --- | --- |
| - mandatory? |  |  |  |  |  |
| - non-mandatory (rotavirus, meningococcal, pneumococcal)? |  |  |  |  |  |

1. Did you have measles-mumps-rubella vaccination before pregnancy?

- Yes
- No

1. If you answered "No" to the previous question, do you intend to have measles-mumps-rubella vaccination during the puerperium?

- Yes
- No

1. Did you have chickenpox vaccination before pregnancy?

- Yes
- No

1. Did you have/are you going to do the scheduled vaccinations in pregnancy: flu?

- Yes
- No

1. Did you have/are you going to do the scheduled vaccinations in pregnancy: diphtheria-tetanus-pertussis?

- Yes
- No

1. Did you have/are you going to do the scheduled vaccinations in pregnancy: COVID-19?

- Yes
- No

1. If you traveled to critical areas during pregnancy, did you seek or were you provided with information regarding vaccine-preventable infectious diseases?

- Yes
- No

# **SECTION 8: Type of job and occupation**

1. Type of job:

| **TYPE OF JOB:** | | |  | |
| --- | --- | --- | --- | --- |
| - **AUTONOMOUS**   (if yes, add a cross "X" in the type of self-employment) | **Yes** | **No** |  |  |
| - Occasional work |  |  |  |  |
| - VAT number |  |  |  |  |
| - Coordinated and continuous collaboration contract |  |  |  |  |
| - **EMPLOYEE**   ((if yes, add a cross "X" in the type of self-employment) | **Yes** | **No** | **If yes, specify job title and/or job profile:** | **If yes, specify the level of classification recognized in the payroll:** |
| - Worker |  |  | (e.g., bookkeeper/ cashier/ administrative activities clerk/ call center operator/ etc.) |  |
| - Clerk |  |  |  |  |
| - Middle manager |  |  |  |  |
| - Manager |  |  |  |  |

1. Work predominantly performed at:

- Corporate office
- Home or away from corporate office
- Mixed home/corporate office
